# Salivary lactoferrin levels decrease in Alzheimer's disease patients

**DOI:** 10.1002/alz.088680

**Published:** 2025-01-09

**Authors:** Saowalak Bunprakop, Chanida Ruchisrisarod, Watayuth Luechaipanit, Thiravat Hemachudha, Poosanu Thanapornsangsuth

**Affiliations:** ^1^ Thai Red Cross Emerging Infectious Diseases Health Science Centre, King Chulalongkorn Memorial Hospital, Bangkok Thailand; ^2^ Faculty of Medicine, Chulalongkorn University, Bangkok Thailand; ^3^ Division of Neurology, Department of Medicine, Faculty of Medicine, Chulalongkorn University, Bangkok Thailand

## Abstract

**Background:**

Alzheimer’s disease (AD) is referred as one of the most common causes of dementia and frailty. To address this impending public health crisis, there is a critical need to identify simple and reliable biomarkers for early AD diagnosis. Recent research has highlighted the potential utility of salivary lactoferrin (Lf) as a promising biomarker for AD diagnosis. In this study, we aim to assess salivary Lf levels in early‐stage AD patients in comparison to nondemented elderly controls with the goal of further elucidating the diagnostic utility of this biomarker, Lf may be a good biomarker candidate for initial and clinically.

**Method:**

A total of 212 patients were included in the study. Saliva samples were collected from nondemented elderly controls (n = 151) and early‐stage AD patients (n = 61). The diagnosis of AD was made clinically using the current guidelines (Jack et al., 2018; Dubois et al., 2021). All patients were diagnosed by PET or plasma phosphorylated tau 181 (P‐tau181), 71 patients including clinical and the Clinical Dementia Rating (CDR). Salivary Lf levels were compared between the two groups and the diagnostic performance of Lf was evaluated in these early‐stage patients.

**Result:**

Among 212 participants included, 139 (65.6%) were female. The median age was 67 years. Salivary Lf levels decreased significantly among early‐stage AD patients (9.02 ug/ml) compared to controls (26.07 ug/ml; P<0.001). Salivary Lf showed moderate correlation with plasma P‐tau181 (Spearman Rho=‐0.472, P<0.001) (Figure 1). For diagnosing AD in early‐stage patients, salivary Lf levels demonstrated an area under the curve of 0.88 (95% confidence interval 0.83‐0.93).

**Conclusion:**

The main findings of this study are that salivary Lf levels decreased significantly in early‐stage AD patients in comparison to nondemented elderly controls, diagnostic performance of salivary Lf is substantial. The hypothesis that immune alterations and brain infections might play an initial role in the development of AD pathology. our hypothesis would determine salivary Lf would be an easy and cheap biomarker that could potentially make it an attractive option for population‐wide screening and early identification of undiagnosed individuals with AD in its early stages.

